# Population pharmacokinetic and pharmacodynamic analysis of rivaroxaban in real-world patients with pulmonary embolism in China

**DOI:** 10.1016/j.rpth.2026.106618

**Published:** 2026-04-30

**Authors:** Jianwei Ren, Yiyao Li, Xin Zheng, Zhen Wu, Juhong Shi, Xiaohong Han

**Affiliations:** 1 Department of Clinical Laboratory, Peking Union Medical College Hospital, Chinese Academy of Medical Sciences & Peking Union Medical College, Beijing, China; 2 Clinical Pharmacology Research Center, Beijing Key Laboratory of Key Technologies for Early Clinical Trial Evaluation of Innovative Drugs for Major Diseases, Peking Union Medical College Hospital, Chinese Academy of Medical Sciences & Peking Union Medical College, Beijing, China; 3 Department of Internal Medicine, Peking Union Medical College Hospital, Chinese Academy of Medical Sciences & Peking Union Medical College, Beijing, China; 4 Department of Pulmonary and Critical Care Medicine, Peking Union Medical College Hospital, Chinese Academy of Medical Sciences & Peking Union Medical College, Beijing, China

**Keywords:** pharmacokinetics, pulmonary embolism, rivaroxaban, pharmacodynamics, precision medicine

## Abstract

**Objectives:**

This study aimed to perform a population pharmacokinetic and pharmacodynamic analysis for rivaroxaban in real-world patients with pulmonary embolism for personalized medication.

**Methods:**

A prospective cohort study was conducted, and 187 eligible patients were included in this study. The analyses were executed using nonlinear mixed-effects modeling, and Monte Carlo simulations were used to obtain individualized dose adjustment strategies.

**Results:**

A total of 517 rivaroxaban plasma concentrations and 376 prothrombin time assays were used for model development. The pharmacokinetic parameters were adequately described using an oral 1-compartment model with first-order adsorption and elimination. The pharmacodynamic model revealed a linear relationship between rivaroxaban plasma concentrations and prothrombin time. The estimated typical values of Ka, V/F, and CL/F were 1.25/h, 53.0 L, and 6.13 L/h, respectively. The creatinine clearance and age were identified as covariates for the rivaroxaban pharmacokinetic profiles. Simulation results showed that patients with moderate renal impairment exhibited an ∼30% increase in rivaroxaban exposure, and that a low dose of 15 mg every day made their exposure similar to that of patients with normal renal function at 20 mg every day.

**Conclusion:**

The developed population pharmacokinetic and pharmacodynamic model can provide individualized dosing strategies for Chinese patients with pulmonary embolism. A lower dose is recommended for patients with moderate renal impairment to avoid overexposure and bleeding events.

## Essentials

• Evidence regarding individualized rivaroxaban dosing for pulmonary embolism (PE) in China remains limited.
• Data from 187 Chinese patients with PE were analyzed to build a population pharmacokinetic/pharmacodynamic model of rivaroxaban.
• Model-based simulations indicated that a low dose may be preferred for patients with renal impairment.
• The model can provide a reference for individualized dosing adjustment for patients with PE in China.

## 1 Introduction

Pulmonary thromboembolism, commonly referred to as pulmonary embolism (PE), represents a critical manifestation of venous thromboembolism (VTE) with high global morbidity and mortality rates. It is reported that PE ranks as the third leading cause of cardiovascular disease deaths globally, only second to coronary heart disease and stroke, underscoring its public health importance [1]. In European and American countries, the estimated annual incidence of PE ranges from 60 to 120 cases per 100,000 general population, with an in-hospital mortality of 14% [[2], [3], [4]]. The epidemiological data in China remain scarce; it has been traditionally assumed that the PE disease burden in Asian populations is lower than that in European and American populations [[5], [6], [7]]. However, recent studies have reported a substantial increase in the incidence of PE in China, highlighting the emerging public health challenge [8,9].

Rivaroxaban, an oral direct factor Xa inhibitor, is widely used for the prevention and treatment of PE. It is rapidly absorbed after oral administration and reaches a maximum plasma concentration in ∼2 to 4 hours [10]. Rivaroxaban undergoes hepatic and renal dual-mode metabolism, and ∼36% is excreted into the urine via the kidneys in a prototype form, and P-glycoprotein and Breast Cancer Resistance Protein are involved in active secretion [10]. In addition, ∼46% of rivaroxaban is enzymatically metabolized, with 18% by cytochrome P450 (CYP) 3A4/A5, 14% by CYP2J2, and 14% by hydrolysis of the amide bond with non-CYP routes [11]. This medication demonstrates considerable interindividual variability in metabolism and elimination, especially in the elderly, those with impaired renal function, and those who require polypharmacy, which leads to variation in pharmacodynamic profiles [12]. The ethnic differences may also contribute to this variability. The elevated clearance is observed in Caucasians compared with Asians, which means increased exposure and potentially elevated adverse event rates in Asians [13].

The EINSTEIN-PE study demonstrated the noninferiority of rivaroxaban vs warfarin in the treatment and prevention of PE. However, these patients were required to meet strict eligibility criteria, and patients at higher risk for adverse events can possibly be recruited in the real world. There is no unified recommendation in routine clinical practice regarding rivaroxaban dosage adjustment for diverse patients, especially when adverse events occur. Notwithstanding the fact that rivaroxaban generally does not require routine coagulation monitoring in most cases, for some special groups, there is a clinical need to assess their anticoagulant activity, further to provide a personalized dosing regimen to balance bleeding and recurrence risks [[14], [15], [16], [17], [18], [19]].

Pharmacokinetic (PK) and pharmacodynamic (PD) profiles can guide personalized medication. Population PK/PD profile analysis uses a nonlinear mixed-effects modeling approach to describe the relationship between fixed and random effects, accordingly estimating population typical values and interindividual and intraindividual variations. This approach can identify and quantify covariate factors that influence PK/PD parameters, thereby enabling the safety and efficacy evaluation of drugs in diverse groups. So far, several research studies have been reported on rivaroxaban PK/PD profiles, but most of them have been performed in Caucasians. Taking the potential racial disparities into account [13], these findings may not be applicable to Chinese patients. Furthermore, current rivaroxaban medication safety studies have focused on patients with nonvalvular atrial fibrillation (NVAF), while those with PE receive less attention [[20], [21], [22]]. Therefore, there is little information on the safety and efficacy of rivaroxaban in real-world patients with PE in China. Despite the gap between PK modeling and clinical application, such models hold great potential for improving dose optimization and provide a reference for guideline development in the future.

In this study, a prospective cohort study was conducted to develop a population PK/PD model to figure out PK/PD profiles of rivaroxaban in Chinese patients with PE, assess potential factors influencing its PK/PD behavior in Chinese patients, and provide tailored dosage adjustment strategies for Chinese patients with PE.

## 2 Methods

### 2.1 Study design

This was a prospective cohort study carried out at Peking Union Medical College Hospital in China from April, 2021 to August, 2024. This study conformed to the Declaration of Helsinki and was approved by the Peking Union Medical College Hospital Institutional Review Board (the approved number: ZS-2796). This study was registered in the ClinicalTrials.gov database with registration number NCT06194617. Informed consent was obtained from all participants in advance.

Inclusion criteria were as follows: (1) patients aged 18 years and older; (2) patients with objectively diagnosed acute symptomatic PE (with or without concurrent deep vein thrombosis) by imaging; (3) patients who completed acute anticoagulation and entered the anticoagulation maintenance phase; (4) patients whose life expectancy was >3 months; (5) patients who met the indications for Xa factor inhibitor use; and (6) patients who were willing to participate in this study, sign the informed consent form, and adhere to regular follow-ups. The acute anticoagulation phase is defined as the initial (21 days) following the diagnosis, during which patients typically receive an intensified dosing regimen, and the “anticoagulation maintenance phase” refers to the subsequent period (from day 22 onward), aimed at secondary prevention, where patients transition to the standard maintenance dose.

Exclusion criteria were as follows: (1) patients who had moderate or severe hepatic impairment (Child-Pugh class B or C); (2) patients who had severe renal impairment (creatinine clearance [CrCL] <15 mL/min); (3) pregnant or breastfeeding women; (4) patients who had spontaneous bleeding tendencies, such as coagulation disorders or low platelet count (<20 × 10^9^/L); (5) patients who had contraindications to other Xa factor inhibitors usage; and (6) patients diagnosed with hereditary thrombophilia and antiphospholipid syndrome.

The eligible patients were administered rivaroxaban with a meal for at least 5 days, and the dosage was determined by their physicians. The demographic information and laboratory test results of eligible patients were prospectively obtained from the medical records, including height, weight, age, gender, albumin level, hematocrit value, hemoglobin concentration, alanine aminotransferase (ALT) level, comorbidities, concomitant medications, serum creatinine (SCr) level, total bilirubin level, and direct bilirubin level. Notably, all baseline laboratory tests were obtained within 24 hours of the confirmed acute PE diagnosis. In addition, the CrCL value was calculated using the Cockcroft-Gault formula [23]. Renal function was categorized based on CrCL values estimated using the Cockcroft-Gault equation as follows: normal (≥90 mL/min), mild impairment (50-89 mL/min), moderate impairment (30-49 mL/min), and severe impairment (15-29 mL/min).

### 2.2 Bioassay

The sparse sampling method was used, and 2 to 4 blood collection points were collected from each enrolled patient, distributed as evenly as possible between the two dosing intervals. The whole blood samples were collected into trisodium citrate-containing tubes predose (within 0.5 hours), 2 ± 0.5, 4 ± 0.5, 6 ± 0.5, and 12 ± 0.5 hours after administration to cover peak and trough concentrations. The whole blood was immediately centrifuged at 3000 g for 10 minutes at 20 °C to obtain plasma. The coagulation testing and drug concentration analysis were performed within 3 months, and all samples were stored at −80 °C until testing.

A sensitive and validated ultrahigh-performance liquid chromatography tandem mass spectrometry method was used to analyze rivaroxaban in plasma [12]. Briefly, plasma samples were protein precipitated and then injected into a Shimadzu LC-20AD UPLC instrument coupled to a Sciex API 4000 mass spectrometer for analysis. The transitions were m/z 436.0 → 145.0 for rivaroxaban and m/z 440.0 → 144.9 for its isotope internal standard. The method was linear in the range of 1 to 500 ng/mL with the lower limit of quantitation of 1 ng/mL. The interday and intraday accuracy were in the range of 88.7% to 106.8%, and interday and intraday precision were < 8.5%.

The prothrombin time (PT) was assayed with Thromborel S Reagent (Siemens) using a Sysmex CS5100 system. The measurements were carried out in strict accordance with the manufacturer’s instructions. The within-run and interrun precision was < 3.5% [24].

### 2.3 Population pharmacokinetics modeling

Sparse concentration datasets were processed with a nonlinear mixed-effects modeling using Phoenix WinNonlin 8.3 (Certara). The first-order conditional estimation extended least squares was employed to estimate PK parameters, including absorption rate (Ka), the apparent clearance (CL/F), and the apparent volume of distribution (V/F) [25]. The model selection and evaluation were performed based on the objective function value (OFV) and diagnostic goodness-of-fit (GOF) plots, including conditional weighted residuals (CWRES) vs time after last dose, CWRES vs population predicted values, observed values vs individual predicted values, and observed values vs population predicted values. The bootstrap and visual predictive check were executed to evaluate the robustness and predictive performance of the final model, respectively.

An attempt was made to fit the plasma drug concentration data using a 1-compartment model and a 2-compartment model, and the basic model was chosen based on a comprehensive consideration of multiple criteria, including GOF plots, OFV decrease, and precision of parameter estimates (calculated as coefficient of variation, CV). The interindividual variability was evaluated using an exponential model. The residual variability was modeled with a proportional model. The equations were as follows:

(1) $$\begin{array}{c} P_{i}=P_{pop}\times e^{\eta_{i}} \end{array}$$

where *P*_*i*_ represented the individual parameter estimate of the *ith* patient, *P*_*pop*_ represented the typical population parameter estimate, and *η*_*i*_ was normally distributed with a mean of 0 and variance of ω^2^.

(2) $$\begin{array}{c} Y=IPRED\times \left(1+\varepsilon \right) \end{array}$$

where *Y* represented the observation, *IPRED* represented the individual predicted value, and *ε* represented the proportional error component, which was assumed to be normally distributed with a mean of 0 and variance of σ^2^.

Multiple covariates were searched using the stepwise method, including height, weight, gender, age, concomitant medication, hemoglobin concentration, hematocrit value, ALT level, albumin level, total bilirubin level, direct bilirubin level, SCr level, and CrCL, which represented individual characteristics, liver and kidney function, and metabolism. Categorical covariates were tested with a scale model:

(3) $$\begin{array}{c} P_{i}=P_{pop}\times \left(1+\theta \times COV\right) \end{array}$$

While continuous covariates were tested using a proportional model:

(4) $$\begin{array}{c} P_{i}=P_{pop}\times {\left(\frac{COV}{{COV}_{median}}\right)}^{\theta } \end{array}$$

where COV and COV_median_ represented the individual and median values of a covariate, respectively, and θ represented the estimated value of the covariate effect.

The *P* value < .01 (OFV reduction > 6.635) for forward inclusion and the *P* value < .001 (OFV reduction > 10.828) for backward elimination were considered as the screening criteria [26]. Furthermore, in the case of continuous variables, when the alterations in PK parameter estimates calculated using the maximum or minimum of the covariates were < 20%, the influence of covariates was considered as not clinically significant. The final covariate model also depended on a comprehensive consideration of multiple parameters.

### 2.4 Population PD modeling

A sequential PD modeling strategy was performed using Phoenix WinNonlin 8.3 (Certara) due to several advantages [27,28]. The population PK was first developed to obtain estimates of PK parameters, which were then input into the subsequent sequential PD modeling process. Finally, a population PK/PD model was developed to estimate PK/PD parameters simultaneously. Based on previous studies, the linear, near-linear, and nonlinear models were used as the structural models to evaluate the relationship between PT and rivaroxaban plasma concentrations [20,29,30]. The interindividual variability was assessed with an exponential model, and the residual variability was modeled with a proportional model. The development and evaluation criterion of the population PD model was the same as that of PK modeling. The equations were as follows:

(5) $$\begin{array}{c} PT=Baselines+Slope\times C \end{array}$$

(6) $$\begin{array}{c} PT=Baselines+Slope\times C^{hill} \end{array}$$

(7) $$\begin{array}{c} PT=Baselines+Slope\times C^{1-hill\times C} \end{array}$$

(8) $$\begin{array}{c} PT=Baselines\times \left(1-\frac{E_{\max}\times C}{{EC}_{50}+C}\right) \end{array}$$

(9) $$\begin{array}{c} PT=Baselines\times \left(1-\frac{E_{\max}\times C^{hill}}{{{EC}_{50}}^{hill}+C^{hill}}\right) \end{array}$$

where *Baselines* represented the baseline of PT time, *Slope* represented the change in PT resulting from the change per unit concentration; *C* represented the rivaroxaban plasma concentration; *hill* represented the exponent of C; E_max_ represented the maximum PT time; and EC_50_ represented the rivaroxaban concentration generating 50% of the maximum PT time.

### 2.5 Model-informed precision dosing

The steady-state 24-hour exposure (calculated as area under the drug concentration-time curve, AUC) values were estimated using Monte Carlo simulations (n = 1000) based on the final model, as well as the virtual Caucasian patients with VTE from a previous report [31]. Regarding renal function levels, in accordance with the label insert for rivaroxaban, the CrCL value was set at 80, 50, and 30 mL/min to represent patients with normal, mildly impaired, and moderately impaired renal function, respectively. The 2-dose regimens were considered equivalent in terms of PK parameter exposure when the AUC ratio and its 90% confidence interval (CI) were between 80% and 125% [[32], [33], [34]]. It was expected to identify potential racial disparity and provide a basis for dose adjustment by exposure simulation. Additionally, the AUC ratio was calculated using the following equation.

(10) $$\begin{array}{c} AUC=\frac{DOSE\left(\text{mg}\right)}{\text{CL}/F\left(L/h\right)} \end{array}$$

It has been reported that abnormal plasma rivaroxaban concentrations are associated with adverse events [35]. In 2018, the International Council for Standardization in Haematology issued the expected direct oral anticoagulant peak and trough concentration ranges in patients with PE/VTE [36]. For rivaroxaban, when used to treat PE/VTE, the expected peak and trough concentrations were 189 to 419 and 6 to 87 ng/mL, respectively. Therefore, Monte Carlo simulations with 1000 subjects were performed to calculate the probability of target attainment (PTA) for both peak and trough concentrations to fall within the expected range. The PTA represented the rationality of the dose regimen. A dose regimen with the highest PTA might be optimal for patients.

Moreover, the effect of covariates on PD was also assessed through Monte Carlo simulations. The average steady-state PT time (PT_ave,ss_) was utilized to represent PD. The average steady-state concentration (C_ave,ss_) was used to calculate PT_ave,ss_ in accordance with the following equations:

(11) $$\begin{array}{c} {PT}_{ave,ss}=f\left(C_{ave,ss}\right) \end{array}$$

(12) $$\begin{array}{c} C_{\text{ave},ss}={AUC}_{24,\text{ss}}/24\;h \end{array}$$

## 3 Results

### 3.1 Study population

A total of 187 patients with PE were enrolled in this study. Of these, ∼35.3% were male, and 41.7% presented with mild to moderate renal impairment. The daily dose of rivaroxaban ranged from 5 mg to 20 mg every day, with the majority (87.7%) receiving either 10 mg every day or 20 mg every day. The median age of the cohorts was 63 years, with 48.1% of patients aged 65 years and older. Comorbidities were prevalent among the participants, and the primary concomitant medications included statins, hypoglycemic agents, and antihypertensive drugs. The detailed demographics of eligible patients were summarized in Table 1. Notably, at least 2 samples were successfully collected from every single patient, and no enrolled patients were excluded from the study for insufficient sample size. The disparity between the total number of rivaroxaban samples (517) and PT samples (376) was due to inadequate residual plasma volume.

**Table 1:** Baseline patient characteristics.

Characteristics	Median (range)/count (%)	Mean ± SD
Number of patients (male/female)	187 (66/121)	
Number of rivaroxaban concentration assays	517	
Number of PT assays	376	
Age (y)	63 (21-92)	58.8 ± 15.8
Age <65 y	97 (51.9%)	
Age ≥65 y	90 (48.1%)	
Age ≥75 y	24 (12.8%)	
Body weight (kg)	70 (49-124)	70.8 ± 14.7
Height (cm)	164 (153-192)	166.2 ± 7.8
Daily dose		
5 mg every d	7 (3.7%)	
10 mg every d	64 (34.2%)	
15 mg every d	16 (8.6%)	
20 mg every d	100 (53.5%)	
SCr level (μmol/L)	68 (36-155)	71.1 ± 19.5
CrCL value		
≥80 mL/min	109 (58.3)	
50-79 mL/min	64 (34.2%)	
30-49 mL/min	14 (7.5%)	
HGB concentration (g/L)	132 (84-177)	133.2 ± 15.5
HCT value (%)	39.5 (24.1-52)	39.7 ± 4.4
ALT level (IU/L)	19 (10-157)	25.3 ± 19.1
Alb level (g/L)	43 (32-49)	42.9 ± 3.5
TBil level (μmol/L)	10.7 (4.1-50.4)	12.3 ± 6.9
DBil level (μmol/L)	3.3 (0.9-16.6)	4.0 ± 2.4
PT (s)	13.8 (9.6-31.9)	14.3 ± 3.0
Comorbidities		
Hypertension	30 (16.0%)	
Cancer	43 (22.9%)	
Coronary artery disease	8 (4.3%)	
Diabetes mellitus	19 (10.2)	
Hyperlipidemia	21 (11.2%)	
Combination therapy		
Statins	49 (26.2%)	
Metformin	10 (5.3%)	
Metoprolol	10 (5.3%)	
Aspirin	7 (3.7%)	
Strong P-gp inhibitors	6 (3.2%)	
Strong BCRP inhibitors	3 (1.6%)	

Alb, albumin; ALT, alanine aminotransferase; BCRP, breast cancer resistance protein; CrCL, creatinine clearance; DBil, direct bilirubin; HCT, hematocrit; HGM, hemoglobin; P-gp, P-glycoprotein; PT, prothrombin time; SCr, serum creatinine; TBil, total bilirubin.

### 3.2 Population PK model

A total of 517 rivaroxaban plasma concentration measurements and 376 PT duration assays were used for model development. The PK profile of rivaroxaban in Chinese patients with PE was described using an oral 1-compartment model with first-order absorption and elimination. The estimated typical values of Ka, V/F, and CL/F were 1.25, 53.0, and 6.13 L/h, respectively. The parameter estimates of the final PK model are detailed in Table 2.

**Table 2:** Parameter estimates in population pharmacokinetic (PK) model.

Parameter (unit)	Population PK model		Bootstrap (n = 1000)	
	Estimate	CV%/Shrinkage%	Median	CI, 2.5%-97.5%
Typical value				
Ka (1/h)	1.25	13.5	1.29	0.922-1.58
V/F (L)	53.0	3.91	53.0	49.0-57.1
CL/F (L/h)	6.13	3.18	6.14	5.75-6.52
Age effect on V/F	−0.231	−34.7	−0.240	(−0.391 to −0.070)
CrCL value effect on CL/F	0.270	23.9	0.271	0.142-0.391
Interindividual variability (%)				
Ka (1/h)	-	-	-	-
V/F (L)	12.5	16.8/15.9	12.5	8.69-16.2
CL/F (L/h)	14.6	13.0/6.6	14.7	10.7-18.5
Residual error				
Proportional error (%)	0.221	8.50/28.1	0.220	0.181-0.252

CI, confidence interval; CL/F, apparent clearance; CV, coefficient of variation; Ka, absorption rate; V/F, apparent volume of distribution.

Among the tested residual models, the proportional model showed the preferred OFV value and satisfaction GOF plots, and was therefore selected to describe residual error. Due to the high shrinkage (99.8%) in the interindividual variation of Ka values, the random effect of Ka values among individuals was removed from the model.

The predefined covariates were added to the basic structural model for covariate screening, and there was no obvious multicollinearity among all covariates. In the forward inclusion process, CrCL value and age had an effect on rivaroxaban CL/F value, resulting in a decrease in OFV values of 18.762 and 13.808, respectively. In addition, age was screened to have an effect on Ka and V/F values, which could result in a decrease in OFV values of 7.772 and 10.909, respectively. In the backward elimination process, the effect of age on CL/F values and the effect of age on Ka values were eliminated due to their OFV values decreasing by 5.935 and 1.362, respectively. These findings were consistent with those from patients with NVAF [30] and acute coronary syndrome [37]. The equations were shown as follows:

(13) $$\begin{array}{c} V/F\left(L\right)=53.0\times {\left(\frac{Age}{63}\right)}^{-0.231} \end{array}$$

(14) $$\begin{array}{c} \text{CL}/F\left(L/h\right)=6.13\times {\left(\frac{C\text{rCL}}{88.3}\right)}^{0.270} \end{array}$$

The median age and CrCL value were 63 years and 88.3 mL/min, respectively. The coefficient (θ) of age on V/F value was −0.231, indicating that the V/F value decreased with age. Rivaroxaban typically demonstrated high plasma protein binding and reversibility [38]. In addition, the coefficient (θ) of the CrCL value on the CL/F value was 0.270, illustrating that the CrCL value had a positive effect on the CL/F value. Patients with impaired renal function might require dose adjustment to prevent drug overexposure. As shown in Table 2, the CV% of parameter estimates from the population PK model was within acceptable limits, confirming the model’s accuracy and reliability.

### 3.3 PD model

A linear model was finally used to describe the relationship between PT and rivaroxaban plasma concentration. The interindividual variation was modeled using an exponential model, and the residual error was assessed with a proportional model. The detailed parameter estimates obtained from the PD model were displayed in Table 3. Through stepwise covariate selection, ALT levels showed a noteworthy influence on the slope of PT time, and the equation is shown below:

(15) $$\begin{array}{c} PT=11.3+0.0184\times {\left(\frac{ALT}{19}\right)}^{-0.201}\times C \end{array}$$

**Table 3:** Parameters estimated from the pharmacodynamic (PD) model.

Parameter (unit)	PD model		Bootstrap (n = 1000)	
	Estimate	CV%/shrinkage%	Median	CI, 2.5%-97.5%
Typical value				
Baseline (s)	11.3	0.711	11.3	11.1-11.5
Slope (s/[μg/L])	0.0184	3.20	0.0184	0.0174-0.0191
ALT level effect on slope	−0.201	−26.1	−0.201	(−0.305 to −0.100)
Interindividual variability (%)				
Baseline(s)	0.242	35.4/46.7	0.243	0.0426-0.443
Slope (s/[μg/L])	10.4	30.5/31.7	10.1	3.95-16.3
Residual error				
proportional error (%)	0.0669	7.61/21.0	0.0667	0.0578-0.0766

ALT, alanine aminotransferase; CI, confidence interval; CV, coefficient of variation.

The baseline PT was 11.3 s with a slope of 0.00184 s per 1 μg/L rivaroxaban. The median of ALT was 19 IU/L, and the coefficient (θ) of ALT on slope was -0.201. The precision of the typical value estimates, interindividual variability, and shrinkage values were all within acceptable ranges, confirming the model’s accuracy and reliability.

### 3.4 Model evaluation

**Figure 1:**
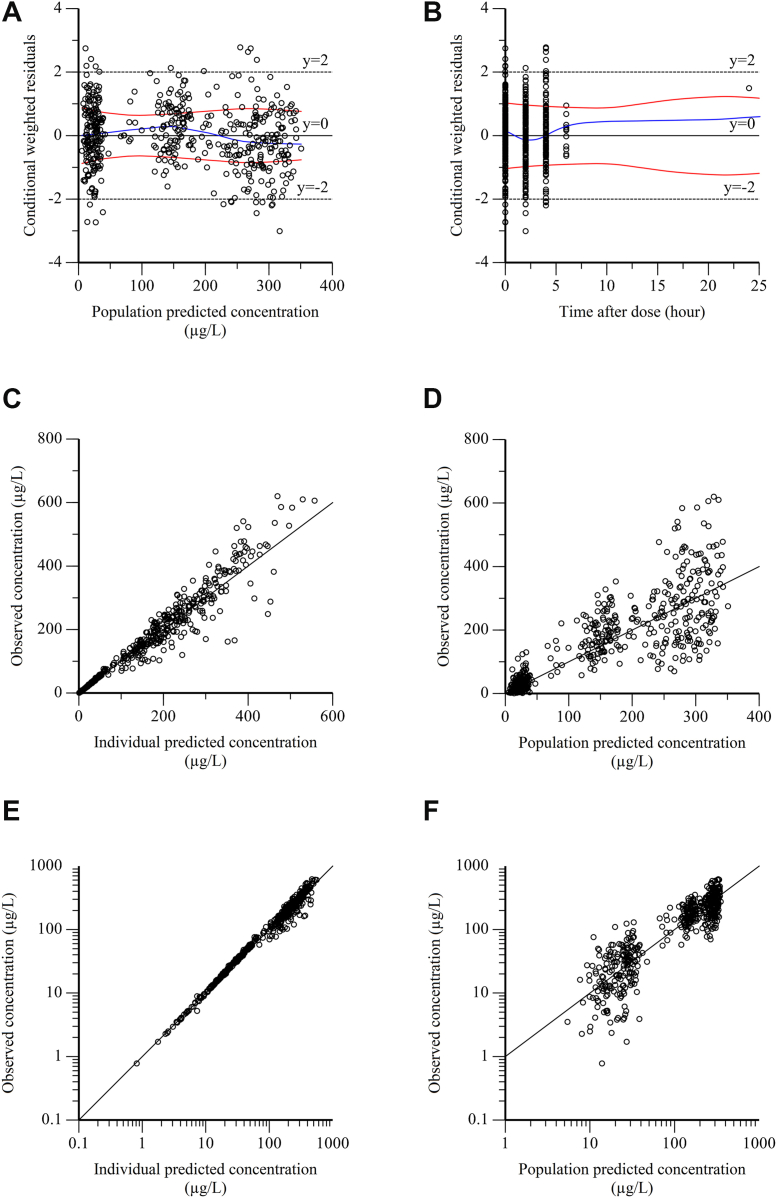
The goodness-of-fit plots of the final population pharmacokinetic (PK) model. (A) Conditional weighted residuals (CWRES) vs population predicted values (PRED). (B) CWRES vs time after last dose (TAD). (C) Observed values (DV) vs individual predicted values (IPRED). (D) DV vs PRED. (E) DV vs IPRED (semi-log scale). (F) DV vs PRED (semi-log scale).

**Figure 2:**
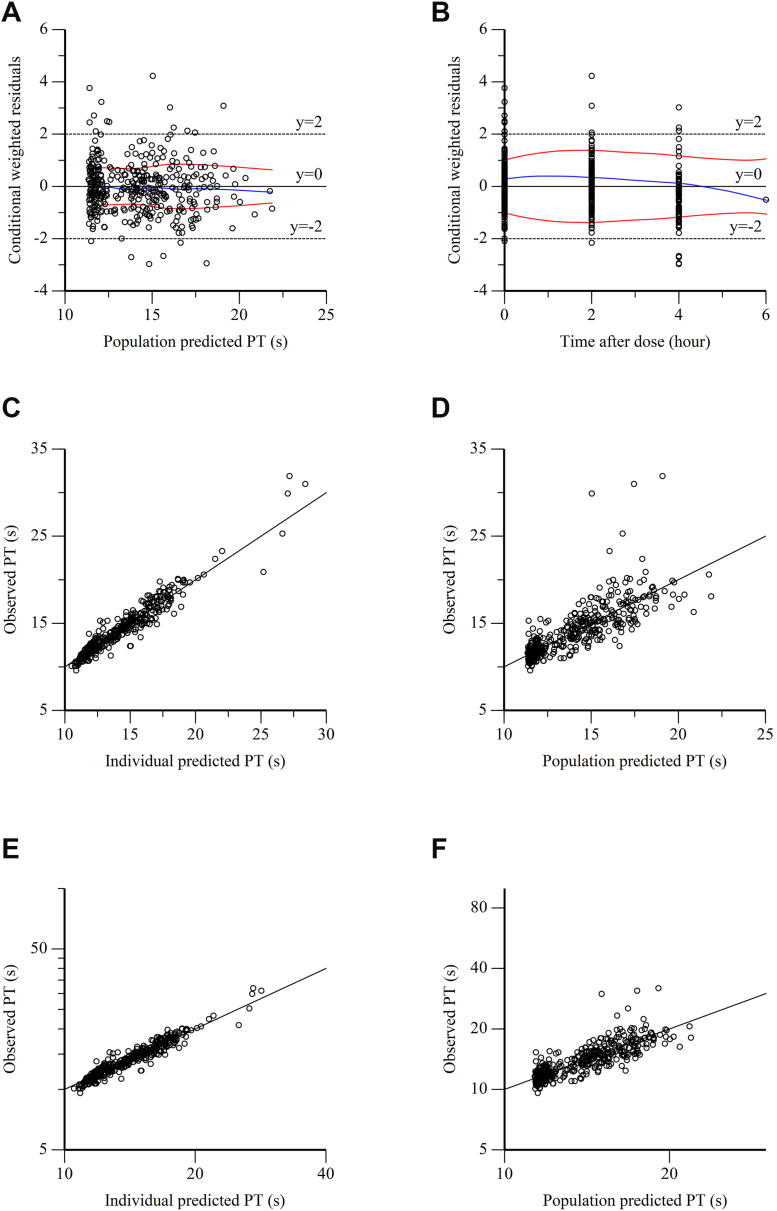
The goodness-of-fit plots of the final population pharmacodynamic (PD) model. (A) Conditional weighted residuals (CWRES) vs population predicted values (PRED). (B) CWRES vs time after last dose (TAD). (C) Observed values (DV) vs individual predicted values (IPRED). (D) DV vs PRED. (E) DV vs IPRED (semi-log scale). (F) DV vs PRED (semi-log scale).

**Figure 3:**
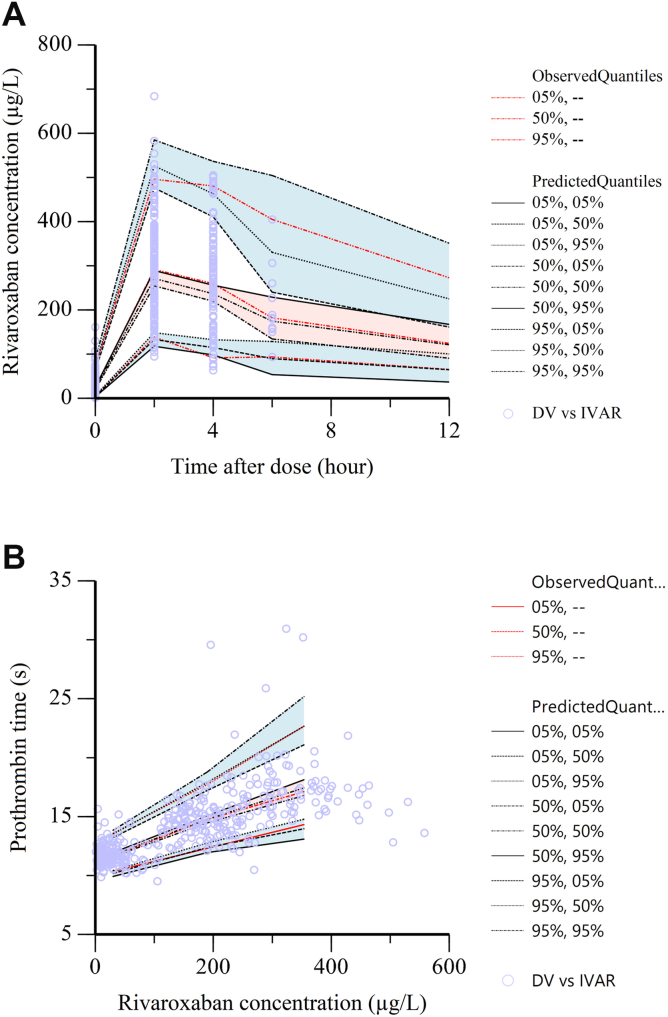
Prediction-corrected visual predictive checks (pcVPC) plots for the population pharmacokinetic (PK) model (A) and PK/pharmacodynamic (PD) model (B). (A) Rivaroxaban concentration vs time; (B) Prothrombin time (PT) vs rivaroxaban concentration. Open circles represent observed data. The red dashed lines indicate the 5th, 50th, and 95th percentiles of the observations. The black lines show the model-predicted 5th, 50th, and 95th percentiles for these red dashed lines. The shaded areas represent the 95% confidence intervals (CIs) of the model predictions (blue for the 5th and 95th percentiles; pink for the 50th percentiles).

The diagnostic GOF plots are shown in Figures 1 and 2. Most CWRES fell within the range of ± 2 (Figure 1A, B), and great agreement was observed between the predicted and observed rivaroxaban plasma concentrations and PT time (Figure 1C–F), demonstrating the robustness of the final model. In addition, as shown in Tables 2 and 3, the simulated PK parameter estimates resulting from bootstrap analysis (n = 1000) were closely aligned with the estimates from the final PK/PD model. The 95% CIs from bootstrap analysis contained the parameter estimates from the final PK/PD model, further supporting that the model accurately describes the observed data. The prediction-corrected visual predictive check plots for the final PK/PD model are illustrated in Figure 3. Most observed plasma concentrations and PT times fell within the 90% prediction intervals, indicating that the final PK/PD model provides reliable predictions.

### 3.5 Model-informed precision dosing

**Figure 4:**
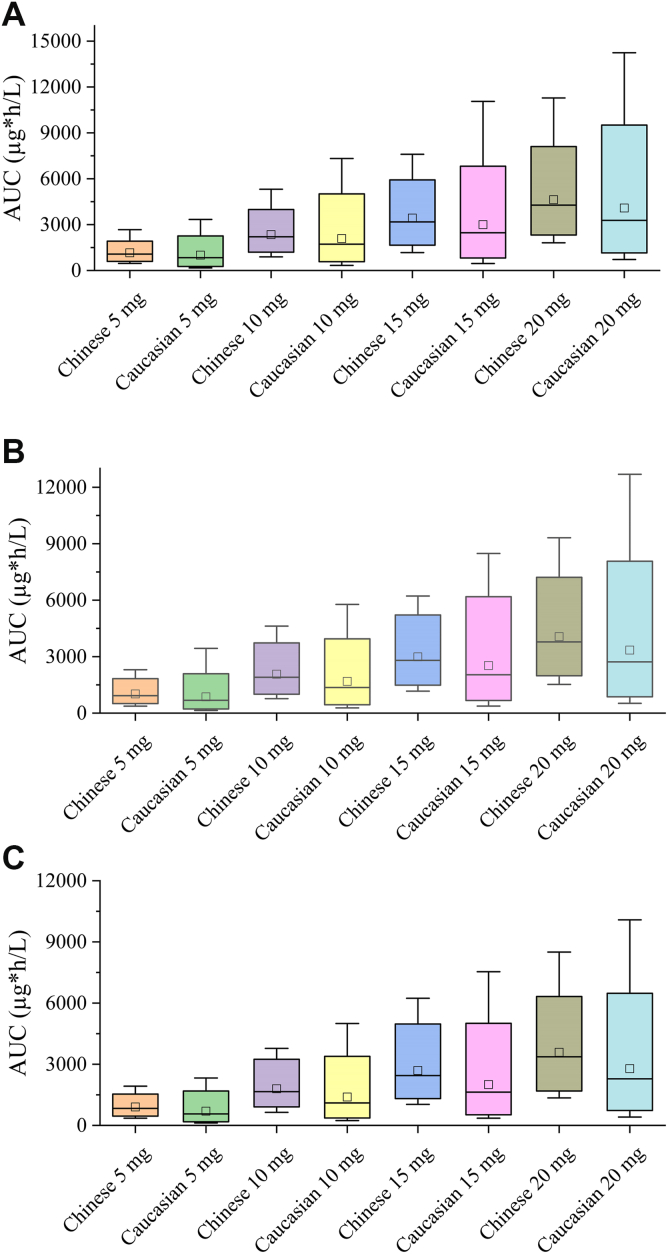
Pharmacokinetic (PK) exposure profiles of Chinese and Caucasian patients under diverse dosing regimens: (A) creatinine clearance (CrCL) value of 30 mL/min, (B) CrCL value of 50 mL/min, and (C) CrCL value of 80 mL/min. The horizontal line and square frame within each box were the mean and median, respectively; the lower and upper limits of the box are the 5th and 95th percentiles, respectively; the lower and upper lines outside the box are the 1st and 99th percentiles, respectively.

PK parameter exposure was simulated in Chinese patients with PE based on the developed model. For Caucasians, due to the lack of direct reports on patients with PE, a population PK study that contained several patients with PE was adopted to simulate the virtual PK parameter exposure [31]. As shown in Figure 4, Chinese patients exhibited increased exposure under similar conditions. For patients with normal renal function (Figure 4C), Chinese patients showed ∼30% higher AUC values compared with Caucasians, which was consistent with findings from Liu et al. [20].

Moreover, the exposure of the Caucasian patients at 20 mg was comparable with that of the Chinese population at 15 mg (2781 vs 2691 μg∗h/L, the ratio 103.3% [90% CI, 99.1%-107.5%]), suggesting that a reduced dose might be appropriate for Chinese patients. For patients with mild and moderate renal impairment (Figure 4A, B), the gap in PK exposure between Chinese and Caucasians narrowed, with increases of ∼20% and 13%, respectively. However, the lack of PD data for the Caucasian population prevents direct dose-reduction recommendations for Chinese patients based solely on these findings. Furthermore, the simulated exposure of Caucasian patients was derived from a virtual population; the results should be interpreted with caution.

For Chinese patients with different renal dysfunction, notable differences occurred in PK exposure. Patients with moderate renal impairment exhibited a ∼30% increase in steady-state AUC values compared with those with normal renal function, suggesting an elevated bleeding risk. In addition, a dose reduction from 20 to 15 mg allowed a similar PK exposure (3425 vs 3585 μg∗h/L, the ratio 95.5% [90% CI, 92.5%-98.5%]). Therefore, 15 mg every day might be optimal for Chinese patients with PE with CrCL values between 30 and 49 mL/min. At the 20 mg dose, rivaroxaban exposure in patients with mild renal impairment was similar to that in normal patients (4059 vs 3585 μg∗h/L, the ratio 113.2%, [90% CI, 110.2%-116.2%]), and a dose reduction from 20 to 15 mg resulted in a 26.4% decrease in exposure, which resulted in a systemic drug exposure that was equivalent to that observed in patients with normal renal function (2986 vs 3585 μg∗h/L, the ratio 83.3%, [90% CI, 80.4%-86.2%]). Therefore, dose adjustment in these patients may depend on peak and trough concentrations.

**Figure 5:**
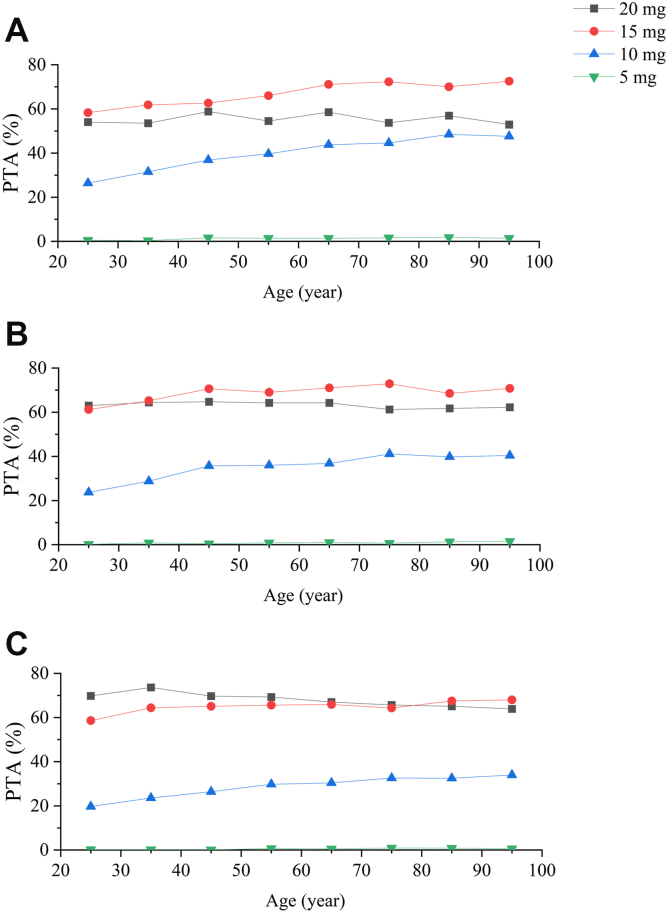
Probability of target attainment (PTA) for peak and trough concentrations within the expected range of (A) creatinine clearance (CrCL) value of 30 mL/min, (B) CrCL value of 50 mL/min, and (C) CrCL value of 80 mL/min.

Initially, therapeutic drug monitoring (TDM) was not required for rivaroxaban. However, with its widespread use, clinical experts have reached a consensus that TDM is beneficial for some special populations, including patients with impaired renal function, extreme body weight, or advanced age. In 2018, the International Council for Standardization in Haematology issued the expected peak and trough concentrations for rivaroxaban in the treatment of PE [36]. Therefore, a series of simulations was performed to evaluate the PTA of peak and trough concentrations of rivaroxaban within the expected range. Additionally, the PTA results were presented in Figure 5. For patients with moderate renal impairment (Figure 5A), the 15 mg every day dosing regimen achieved an optimal PTA. Therefore, the 15 mg every day dosing regimen might be optimal for patients with a CrCL value of 30 to 49 mL/min, which is consistent with the PK parameter exposure simulation results.

For patients with mild renal impairment (Figure 5B), the PTAs of 20 mg and 15 mg were similar in those younger than 35 years. However, the PTA of the 15 mg progressively exceeded that of the 20 mg in those aged 35 years and older, demonstrating that 15 mg every day might be a more favorable option for this population. Furthermore, for patients with normal renal function (Figure 5C), there was a gap between the PTA of 15 mg and 20 mg in patients aged 75 years and below, and the gap gradually diminished with age. The trend was reversed in patients older than 75 years, but the reversed disparity was not obvious. Therefore, dose adjustments might not be required for patients older than 75 years with normal renal function.

**Figure 6:**
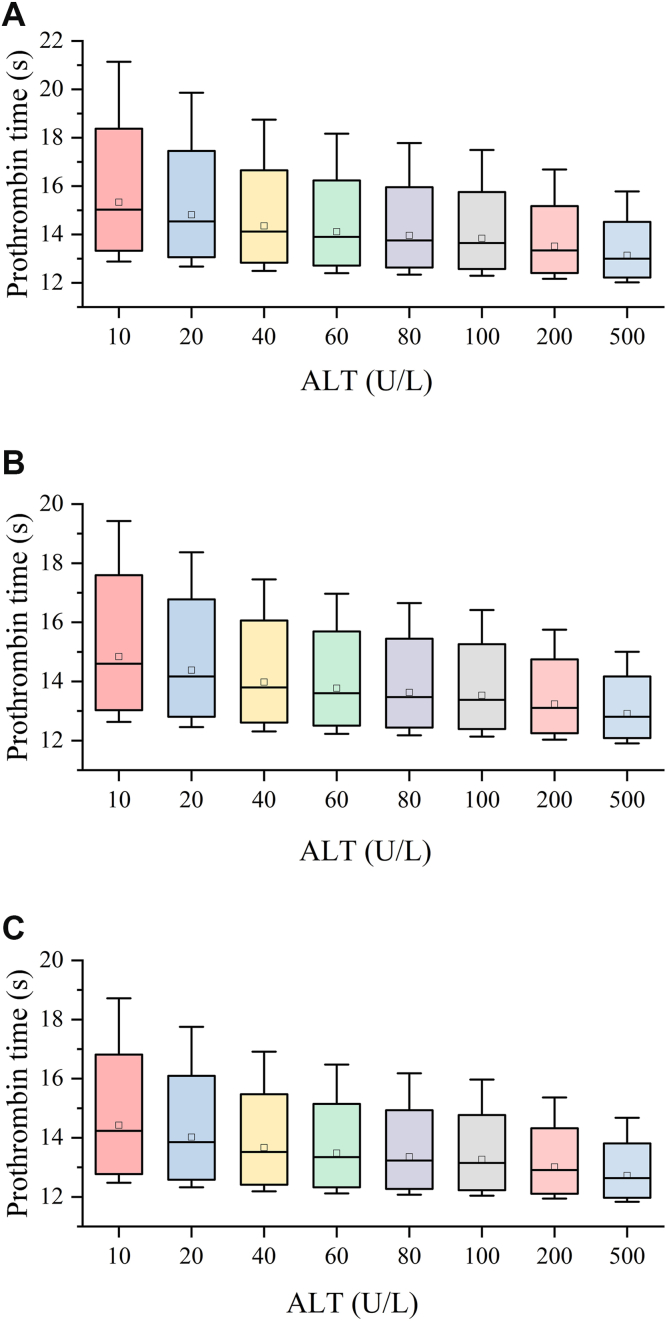
Boxplots of the distribution of simulated average steady-state prothrombin time (A), creatinine clearance (CrCL) value of 30 mL/min (B), CrCL value of 50 mL/min (C), and CrCL value of 80 mL/min. The horizontal line and square frame within each box were the mean and median, respectively; the lower and upper boundaries of the box are the 5th and 95th percentiles, respectively; the lower and upper lines outside the box were the 1st and 99th percentiles, respectively.

In the PD model, ALT level was observed to exert a negative influence on the slope of PT time, serving as an indicator of hepatocyte damage. The average steady-state PT time was simulated at a 20 mg every day dose regimen to assess quantitatively the effect of ALT level on PT time with ALT levels in the range of 10 to 500 IU/L quantitatively, and the results were presented in Figure 6. It was observed that the ALT level did not have a considerable influence on the average steady-state PT time, with a rise in the ALT level from 10 to 157 IU/L resulting in a decrease in PT time by ∼7%. Therefore, the influence of ALT level on the slope of PT time might not be clinically significant. In addition, the patients included in this study had normal or mildly impaired liver function, which typically did not lead to coagulation disorders. The negative effect might be attributed to an increase in coagulation factors in the blood as a result of mild hepatocyte damage.

Following a consolidated evaluation of PK parameter exposure and TDM simulation results, a proposal for rivaroxaban dose adjustment for Chinese patients with PE in the maintenance phase was formulated and presented in Table 4. In summary, (1) a 20 mg every day regimen was appropriate for patients with normal renal function; (2) a 15 mg every day regimen was recommended for patients with moderate renal impairment; (3) for patients with mild renal impairment, age segmentation existed such that a 20 mg every day dose regimen was indicated for patients younger than 35 years, whereas a 15 mg every day dose regimen applied to patients aged 35 years and older. Compared with the approved dose, the adjusted dose was lower for some patients, which was beneficial for reducing bleeding events.

**Table 4:** Rivaroxaban dose adjustment strategies for Chinese patients with pulmonary embolism (PE).

Age (y)	CrCL value (mL/min)		
	≥80	50-79	30-49
25	20 mg every d	20 mg every d	15 mg every d
35	20 mg every d	20 mg every d	15 mg every d
45	20 mg every d	15 mg every d	15 mg every d
55	20 mg every d	15 mg every d	15 mg every d
65	20 mg every d	15 mg every d	15 mg every d
75	20 mg every d	15 mg every d	15 mg every d
85	20 mg every d	15 mg every d	15 mg every d
95	20 mg every d	15 mg every d	15 mg every d

Light green: 20 mg; light yellow: 15 mg.

CrCl, creatinine clearance.

## 4 Discussion

This was the first study to develop a population PK/PD model for rivaroxaban in Chinese patients with PE. The PK profile of rivaroxaban was adequately described by an oral one-compartment model, and the PD information was accurately characterized by a linear model. Attempting to fit alternative multicompartment models to our sparse dataset would inevitably lead to overparameterization and unstable parameter estimates. Therefore, the final one-compartment model should be interpreted as a robust empirical approximation tailored to the available data, rather than a reflection of absolute physiological certainty regarding the multiphase disposition of rivaroxaban. The similar structural models were also used in populations with acute coronary syndrome [37], NVAF [20,30], and deep vein thrombosis (DVT) [39,40], demonstrating the reliability of this study. The developed population PK/PD model demonstrated an excellent fit to the sparse data set, providing a reliable prediction of the rivaroxaban PK profile with robustness and accuracy. The influence of CrCL value on CL/F value, as well as that of age on V/F value, was accurately estimated in the model and aligned with physiological expectations. While comprehensive clinical data regarding concomitant medications were recorded, the limited number of patients receiving strong P-glycoprotein or breast cancer resistance protein inhibitors in our specific cohort precluded the integration of these drug-drug interactions into the final model. Therefore, it was important to acknowledge that the concurrent use of such agents might still account for a portion of the unexplained interindividual variability in rivaroxaban exposure. In addition, a series of simulations was performed to provide a personalized dose adjustment strategy for Chinese patients with PE. In elderly patients, reduced plasma protein secretion can result in low-level bound drug and an increased proportion of free drug in the bloodstream. This elevated free drug concentration may lead to adverse events such as skin bruising, mucosal bleeding, and gastrointestinal bleeding.

In the PubMed database, some rivaroxaban population PK/PD studies were retrieved and listed in Table 5 [20,[29], [30], [31],^39^,^40^]. As shown in Table 5, there were some differences in PK parameters between Caucasian patients with VTE and DVT, and the gap in median age and mean body weight might be the potential reason. Compared with patients with VTE, patients with DVT showed older age (61 vs 52 years), lower weight (80 vs 88 kg), and higher CrCL values, which might explain their higher clearance and volume of distribution. In this study, the patients’ median age (63 years) was closer to that of patients with DVT, but the mean body weight (70 kg) was lower than that of both patients with DVT and patients with VTE. Most importantly, the CrCL values in patients with DVT were 87.4 ± 1.5 mL/min (mean ± SD), while in this study, 58% of patients had a CrCL value of ≥80 mL/min, as did 67% of patients with VTE. Considering that the CrCL value had a greater impact on exposure, patients with VTE were selected ultimately to simulate the virtual exposure of Caucasians.

**Table 5:** Comparison of pharmacokinetic/pharmacodynamic (PK/PD) parameters in diverse populations [20,[29], [30], [31],^39^,^40^]

Parameter (units)	Chinese PE	Chinese DVT	Chinese NVAF	Japanese NVAF	Caucasian VTE	Caucasian DVT	Caucasian NVAF
PK model							
Ka (/h)	1.25	0.617 (fixed)	0.617 (fixed)	0.617	1.21	1.23	1.16
CL/F (L/h)	6.13	5.72	5.04	4.72	8.86	5.67	6.10
Central V/F (L)	53.0	45.4	40.4	42.9	101	54.4	79.7
PD model (PT)							
Baselines(s)	11.3	-	13.9	13.7	-	-	11.4
Slope (s/[μg/L])	0.0184	-	0.0132	0.0227	-	-	0.043

CL/F, apparent clearance; DVT, deep vein thrombosis; Ka, absorption rate; PE, pulmonary embolism; NVAF, nonvalvular atrial fibrillation; PT, prothrombin time; V/F, apparent volume of distribution; VTE, venous thromboembolism.

In this study, the typical CL/F value was similar to that in Chinese patients with DVT, Caucasian patients with DVT, and Caucasian patients with NVAF; slightly higher than that of Chinese patients with NVAF and Japanese patients with NVAF; but lower than that of Caucasian patients with VTE. This trend was roughly negatively correlated with the median age of the patients in each study, which may be the result of reduced drug clearance due to impaired renal function. In addition, the variation may be partially attributable to ABCB1 gene polymorphisms. It was reported that rivaroxaban clearance was 47.6% higher in patients carrying the ABCB1 rs 4728709 AA/GA genotype than in those with the GG genotype [21]. In addition, Caucasians had a higher frequency of ABCB1 rs 4728709 AA/GA than the Asian populations by ∼2.1-fold, which may be the potential reason for the PK parameter discrepancy between Caucasians and Chinese [13].

The typical value of V/F in this study was 53.0 L, which was similar to that of Caucasian patients with DVT, slightly higher than that of Chinese patients with DVTs, patients with NVAF, and Japanese patients with NVAF, but lower than that of Caucasian patients with VTE and patients with NVAF. The height and weight, as the main covariates of V/F values, may be the main reason for the difference in V/F values. As for the Ka value, the 2 existing Chinese studies [20,39] both adopted the Ka value from the Japanese study [29] as a fixed value for subsequent model development, so comparisons were not available across Chinese populations. Interestingly, the Ka values in this study, similar to those of Caucasian patients, were considerably higher than those in Japanese patients and almost twice as high. Rivaroxaban, administered with food, enhanced its contact with the gastric mucosa and prolonged its gastric residence time [38]. Data from the Food and Agriculture Organization of the United Nations indicated that the consumption per capita of vegetables, meat, and other food in Japan was much lower than that in China and the United States [41]. Additionally, the inadequate food intake might limit the absorption of rivaroxaban in Japanese patients. Therefore, it was recommended not to adopt the Ka values from the Japanese study as a fixed value in PK model development for the Chinese patients in future studies.

In the PD model, the typical value of baseline PT time in Chinese patients with PE was similar to that in Caucasian patients with NVAF and slightly lower than that in Chinese patients with NVAF and Japanese patients with NVAF, which may be attributed to the discrepancies in the age composition of the study population as well as the disparities in anticoagulation status, cardiovascular function, and other physiological parameters. However, elevated ALT levels typically reflected liver cell damage, which was an important synthesis site for multiple thrombin, prothrombin, and coagulation factors. The patients included in this study had normal or mild liver impairment, which typically did not affect the synthesis of clotting factors. Hepatocellular injury leads to the premature release of intracellular clotting factors into the bloodstream, which may be the main reason for the negative effect of ALT level on the slope.

For patients with PE with normal renal function, an exposure increase of ∼30% was observed in Chinese patients compared with Caucasians. A similar finding was also reported in patients with NVAF [20]. For patients with normal renal function, comparable exposures occurred in Chinese patients at 15 mg every day and in Caucasian patients at 20 mg every day. Several studies revealed that Asian patients might require a lower dose [13]. Additionally, the results in this study seemed to support this view, but the results should be interpreted with caution due to the limited PD data. Furthermore, the low dose of 15 mg was adequately evidenced to be safe and effective in Japanese patients with NVAF [29,42,43]. Considering the similarity between Chinese and Japanese patients in PK profiles, the approved dose based on the Japanese patients with NVAF might also be applicable to Chinese patients with NVAF. Moreover, renal function exerted a considerable influence on the rivaroxaban exposure, and simulations were stratified by CrCL values with cutoff points of 30, 50, and 80 mL/min, consistent with the rivaroxaban insert label. For patients with PE with a CrCL value of 30 to 49 mL/min, a ∼30% increase in exposure was observed compared with those with a CrCL value of ≥80 mL/min. This increase was lower than that (∼50%) reported in acute coronary syndrome [19] and patients with NVAF [20]. In previous studies, dose adjustment strategies were proposed based on exposure simulation, and the potential of TDM was not fully exploited [20,37,44]. In this study, exposure simulation and TDM were used in combination to form a dosing optimization strategy. The adjusted regimen demonstrated the highest PTA while preventing excessive exposure and was thus recommended for patients with PE. Notably, Table 4 was intended to serve as a therapeutic reference for individualized dose optimization, rather than as absolute clinical guidance.

The PD markers for rivaroxaban typically included PT time, activated partial thromboplastin time, prothrombinase-induced clotting time, Heptest, FXa activity, and partial thromboplastin time. Among them, PT time and Xa factor activity were tightly correlated with plasma rivaroxaban concentrations, as evidenced in previous studies [38,45]. The Xa factor activity assays were regarded as the most appropriate method for quantifying plasma rivaroxaban concentrations [45,46]. However, no robust evidence has demonstrated a direct association between rivaroxaban plasma concentrations and clinical adverse events. Instead, PT had been linked to clinically relevant hemorrhage risk [47], and its prolongation (about 20-30 seconds) typically implied an elevated bleeding risk [48,49]. The absence of clinical relevance for other PD markers limited their application. Consequently, PT time was adopted as the PD marker in this study, which was consistent with prior population PK/PD studies of rivaroxaban [20,30,37,38,40,50]. Notably, unlike conventional anticoagulants, the international normalized ratio and the international susceptibility index, which were derived from PT time, did not apply to rivaroxaban [[51], [52], [53]]. Although the sensitivity of PT time to rivaroxaban is known to be reagent-dependent, interreagent variability was minimized in our study by utilizing a standardized assay system. While severe liver injury is known to impair coagulation factor synthesis and prolong PT time, the vast majority of patients in our cohort had normal or only mildly impaired liver function. The observed marginal decrease in PT time (∼7%) with rising ALT levels may be attributed to the transient leakage of presynthesized coagulation factors from slightly damaged hepatocytes rather than impaired synthesis. Given the minimal magnitude of this change, the influence of a mild elevation of ALT levels on PT time is not considered clinically significant.

In clinical practice, several equations were typically utilized to estimate renal function, which commonly included CrCL values, calculated using the Cockcroft-Gault equation, as well as the estimated glomerular filtration rate based on the Modified Diet in Renal Disease or Chronic Kidney Disease Epidemiology Collaboration equations. There were some inconsistencies between these equations, and the variability might lead to controversial dose adjustment strategies. Compared with CrCL values, the rest might lead to severe misclassification and dosage inappropriateness in 36.2% and 35.8% of patients, respectively [54]. The study by Kubitza et al. [55] demonstrated that CrCL values calculated using the Cockcroft-Gault formula were significantly correlated with rivaroxaban exposure in patients. Compared with subjects with normal renal function, the AUC values of rivaroxaban increased by 1.4-, 1.5-, and 1.6-fold in subjects with mild (CrCL values, 50-80 mL/min), moderate (CrCL values, 30-49 mL/min), and severe (CrCL values, 15-29 mL/min) renal impairment, respectively. Therefore, in this study, the CrCL value was used to estimate renal function, which was calculated using the Cockcroft-Gault equation.

This study was dedicated to providing personalized dose adjustment for Chinese patients with PE in the real world to prevent adverse events, including recurrent thrombosis and clinically relevant hemorrhage. Despite some strengths of this study, it was important to acknowledge some limitations. First, the genotype information of the patients was not available, making it impossible to analyze potential racial discrepancies at the molecular level. Second, the range of concomitant medications was extensive and dispersed, which might explain why concomitant medication was not identified as a covariate with an impact on PK parameters. Third, due to the lack of direct data, the PK/PD parameters of Caucasians were from published literature, which may introduce bias in subsequent exposure simulations. Therefore, the comparisons should be interpreted with caution. In the future, large-scale cohort studies are needed to validate these conclusions in this study. Finally, constrained by the sparse sampling inherent to the real-world clinical setting, the use of a one-compartment model was inevitable. This structural simplification may inadvertently incorporate uncharacterized variance into the population parameters, thereby overestimating the actual interindividual variability to some extent.

## 5 Conclusion

In this study, a population PK/PD model of rivaroxaban was successfully developed for Chinese patients with PE. The CrCL values and age were identified as covariates of the rivaroxaban PK/PD profile. Model-based simulations indicated that a low dose may be preferred for patients with renal impairment. The population PK/PD model can provide individualized dosing strategies for Chinese PE populations.
